# Adjuvant Perioperative Intraperitoneal Chemotherapy in Locally Advanced Colorectal Carcinoma: Preliminary Results

**DOI:** 10.5402/2011/529876

**Published:** 2011-05-22

**Authors:** A. A. K. Tentes, I. D. Spiliotis, O. S. Korakianitis, A. Vaxevanidou, D. Kyziridis

**Affiliations:** ^1^Surgical Department, Didimotichon General Hospital, Konstantinoupoleos 1, 68300 Didimotichon, Greece; ^2^Surgical Department, Mesologgi General Hospital, Greece; ^3^Department of Anesthesiology, Didimotichon General Hospital, Mesologgi, Greece; ^4^Department of Anesthesiology, Mesologgi General Hospital, 68300 Didimotichon, Greece

## Abstract

*Background and Aims*. Intraperitoneal chemotherapy is a basic tool in the treatment of peritoneal malignancy. The purpose of the study is to investigate the effect of adjuvant perioperative intraperitoneal chemotherapy in the treatment of locally advanced colorectal cancer. *Patients and Methods*. Patients with T_3_ and T_4_ colorectal carcinomas that underwent R_0_ resection received either hyperthermic intraoperative intraperitoneal chemotherapy (HIPEC group = 40 patients) or early postoperative intraperitoneal chemotherapy (EPIC group = 67 patients). The survival, the recurrences and the sites of recurrence were assessed. *Results*. The 3-year survival rate for HIPEC group was 100% and for EPIC group 69% (*P* = .011). Nodal infiltration was found to be the single prognostic indicator of survival. The incidence of recurrence in EPIC group was higher than in HIPEC group (*P* = .009). The independent indicators of recurrence were the use of HIPEC and the degree of differentiation (*P* < .05). *Conclusions*. Intraperitoneal chemotherapy, particularly HIPEC, as an adjuvant in locally advanced colorectal carcinomas appears to improve survival and decrease the incidence of recurrence.

## 1. Introduction

The incidence of recurrence for Dukes' B and C colorectal carcinomas is approximately 50% [1]. The corner stone in the surgical treatment of rectal cancer is total mesorectal excision [2]. The incidence of locoregional recurrence has been reduced significantly by total mesorectal excision [3]. Similar to this approach, extensive lymph node resection has been questioned for colon cancer [4]. So far, numerous adjuvant treatments have been used to prevent the development of recurrence and improve survival [5]. Preoperative irradiation in rectal cancer has been shown to significantly reduce the incidence of locoregional recurrences [6], but only one study showed improvement of survival [7]. Systemic chemotherapy has shown to improve survival in stage III colon cancer [8]. Both irradiation and systemic chemotherapy are followed by significant toxicity in contrast to intraperitoneal chemotherapy [8, 9]. Intraperitoneal chemotherapy integrated in cytoreductive surgery is the standard treatment of peritoneal malignancy that which survival by eradicating the microscopic residual tumor [10]. Locoregional recurrence in locally advanced gastrointestinal carcinomas is the result either of tumor involving the serosa and perforation of the bowel wall or of iatrogenic dissemination of cancer emboli which give rise to locoregional tumor within 2-3 years [11]. If chemotherapy is used by the intraperitoneal route, microscopic residual tumor resulting from surgical manipulations in locally advanced colorectal cancer surgery may possibly be eradicated. 

 The purpose of the study is to investigate the effect of adjuvant perioperative intraperitoneal chemotherapy after resection of locally advanced colorectal carcinomas. Hyperthermic intraoperative intraperitoneal chemotherapy (HIPEC) is compared with early postoperative intraperitoneal chemotherapy (EPIC). The end point of the study is the investigation of overall survival, recurrence rate, and sites of recurrence.

## 2. Patients and Methods

From 1999 to 2004 patients with locally advanced (T_3_, T_4_) colorectal carcinomas were prospectively assigned to undergo R_0_ resection and receive EPIC as an adjuvant. From 2005 until today patients with the same tumor characteristics were prospectively assigned to undergo R_0_ resection and receive HIPEC as an adjuvant.

### 2.1. Eligibility Criteria

Patients meeting the following criteria were eligible for treatment: (1) age over 18 years that could undergo major surgery, (2) satisfactory cardiopulmonary function with no evidence of myocardial infarction during the previous 6 months, (3) normal liver function, (4) normal renal function (blood urea < 50 mg/dl and creatinine level < 1.5 mg/dl), (5) normal white blood cell count (>4000) and platelets (>150.000), and (6) performance status >50% according to Karnofsky performance scale. 

 The exclusion criteria were the following: (1) age under 18 years, (2) the presence of irresectable metastatic disease, (3) previous treatment for cancer, (4) the presence of a second malignant tumor at high risk for recurrence (except for skin basal cell carcinoma or in situ cervix carcinoma radically treated), (5) Karnofsky performance status <50%, (6) psychosis, drug or alcohol addiction, (7) the presence of diffuse peritonitis, and (8) pregnancy.

### 2.2. Definitions and Diagnosis

Right colon was considered the segment of the large bowel, proximal to the left colic flexure. Left colon was considered the segment of the large bowel from the left colic flexure distal to the peritoneal reflexion. The rectum was considered the segment of the large bowel, distal to the peritoneal reflexion.

The diagnosis of carcinoma was established by physical examination, biochemical and hematological examinations, tumor markers (CEA, CA 19-9, and CA-125), abdominal and thoracic CT scan, whole-body bone scan, colonoscopy, and tumor biopsy.

The protocols were approved by the ethical committee of the hospitals and the patients signed informed consent.

### 2.3. Treatment

Samples for peritoneal cytology were taken after abdominal exploration. Total mesorectal excision was required for tumors of the middle and lower rectum. The assessment of sphincter preservation was on the surgeon's judgment.

The Coliseum technique (open abdomninal method) was used during HIPEC which was administered before the reconstruction of the continuity of the alimentary tract for 90 min if mitomycin-C (15 mg/m^2^) was used and for 60 min if oxaliplatin (130 mg/m^2^) was used. The temperature during perfusion was maintained at 42.5–43°C. Oxaliplatin or mitomycin-C was used according to surgeon's preference. The cytostatic drug was diluted in 2 liters of normal saline for HIPEC and in 1–1.5 liters of D_1.5_W if EPIC was used.

EPIC was given through a Tenckhoff catheter that was positioned at the tumor bed for the first 5 postoperative days. The chemotherapy regimen was instilled rapidly in the peritoneal cavity and dwelled for 23 hours. Then the drains were opened for one hour, and instillation of the regimen began again. 5-FU (600 mg/m^2^) with 50 meq NaHCO_3_ was used for EPIC.

All the patients who were pTNM stage III and IV received additional 6 cycles of systemic chemotherapy with 5-FU + leukovorine.

 Right colon carcinomas were treated with right or transverse colectomy depending on tumor location. Left colon carcinomas were treated with left colectomy and rectal tumors with low anterior resection or abdominoperineal resection. A protective colostomy was always used if low anterior resection was performed.

Toxicity was recorded from the time of operation and during followup.

### 2.4. Histopathology

All the specimens were histopathologically examined. Details about T, N, TNM stage, degree of differentiation, and circumferential margins of resection were recorded.

### 2.5. Followup

Followup was possible every 4 months during the first year and every 6 months later by physical examination, hematological and biochemical examinations, tumor markers (CEA, CA 19-9, CA-125), thoracic and abdominal CT scan, and whole-body bone scan whenever it was indicated. Colonoscopy was performed once a year after the first year of followup. The recurrences and the sites of recurrence were recorded.

### 2.6. Statistical Analysis

The proportions of patients with a given characteristic were compared by chi-square analysis or by Pearson's test. Differences in the means of continuous measurement were tested by the Student's *t*-test. The survival curves were obtained using the Kaplan-Meier method, and the comparison of curves was calculated using the log-rank test. Cox regression analysis made multiple analysis of survival possible. Logistic regression analysis made multiple analysis of recurrence, morbidity, and mortality possible. A two-tailed *P* value <.05 was considered statistically significant.

## 3. Results

The HIPEC-group consisted of 45 patients and the EPIC group of 63 patients. Five patients from the HIPEC-group and 6 patients from the EPIC group were rejected from the analysis because they were found to have pT_2_N_0_M_0_ tumors or they had peritoneal malignancy. The groups were comparable for age, gender, T stage, nodal status, TNM stage, anatomic distribution of the tumor, type of surgical operation, morbidity, and sites of recurrence. They were different in performance status, degree of differentiation, and recurrence (Table 1). In 2 patients of HIPEC-group, liver metastatic disease was detected intraoperatively. The lesions were completely resected, and the patients were included in the study protocol. All the surgical operations were R_0_ resections. All the samples for peritoneal cytology were negative for malignant cells.

### 3.1. Morbidity and Hospital Mortality

EPIC was found to be related to hospital mortality (*P* = .034) but it did not influence the survival independently. The hospital mortality was 10.3%. Only one patient in HIPEC-group died in the immediate postoperative period (Table 1). The overall morbidity rate was 39.2%. The postoperative complications are listed in Table 2. No clinical variable was found to be related to morbidity.

### 3.2. Survival

**Figure 1:**
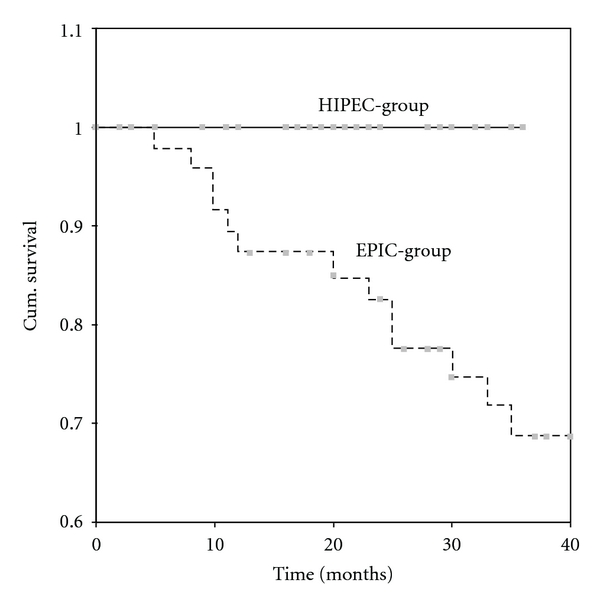
Survival according to type of intraperitoneal chemotherapy (continuous line-HIPEC-group, dotted line = EPIC group).

The 3-year survival rate for the HIPEC-group was 100%. For EPIC group mean survival was 100 ± 6 months, and 3-year survival rate was 69% (*P* = .011) (Figure 1). Nodal status was also found to be related to survival (*P* = .0262). Multivariate analysis of survival showed that only nodal status was a prognostic indicator of survival (HR = 5.221, *P* = .022, 95% CI = 1.118–4.182).

### 3.3. Followup

The median followup time for HIPEC and EPIC group was 17 and 28 months, respectively. During followup, one patient (2.5%) in the HIPEC-group was recorded with recurrence and 16 (28%) patients in the EPIC group (*P* = .009). The patient in the HIPEC-group had colon cancer and developed liver metastasis. In the EPIC group 3 patients with rectal cancer developed recurrence and only one of them locoregional. Perioperative intraperitoneal chemotherapy (*P* = .001) and the degree of differentiation (*P* = .024) were found to be related to recurrence. By multivariate analysis the degree of differentiation (HR = 5.658, *P* = .017, 95% CI  =  .006–.516) and the use of intraperitoneal chemotherapy (HR = 6.663, *P* = .001, 95% CI  =  .008–.519) were found to be prognostic indicators for the development of recurrence.

## 4. Discussion

Patients with locally advanced gastrointestinal carcinomas are at high risk for recurrence. Locoregional recurrences are usually detected at the resection site and the closest peritoneal surfaces [11]. Cancer emboli from traumatized interstitial tissues, severed lymphatic vessels, and venous blood loss implant adherently at the resection site and in abraded peritoneal surfaces. Platelets, polymorphonuclear cells, and monocytes infiltrate the fibrinous exudate that accumulates during wound healing. Growth factors are released to stimulate fibroblast proliferation and local collagen production. Growth factors modulating wound healing promote cancer proliferation at the site of wound healing. This is particularly true for tumors located in narrow limits of resection. Cancer emboli grow rapidly and give rise to detectable tumors within 2-3 years after initial surgical operation [11].

Numerous publications for pseudomyxoma peritonei [12], colorectal cancer [9], gastric cancer [13], peritoneal mesothelioma [14], and ovarian cancer [15] have shown that microscopic residual tumor after cytoreductive surgery combined with intraperitoneal chemotherapy in peritoneal malignancy may be completely eradicated. The success of the treatment mainly depends on the completeness of cytoreductive surgery and on the extent of the peritoneal malignancy [16]. Intraperitoneal chemotherapy is effective on tumor emboli that are less than 2-3 mm in their largest diameter. The peritoneal-plasma barrier has the property of delaying the absorption of macromolecular substances to the systemic circulation [17]. Most of the cytotoxic drugs used for intraperitoneal administration are macromolecular substances. The drugs instilled intraperitoneally act longer and intensely at the peritoneal surfaces where cancer emboli are entrapped [18]. The cytotoxic effect is enhanced by heat [19].

Three of the most potent cytotoxic agents for gastrointestinal carcinomas are 5-FU, mitomycin-C and oxaliplatin. All have been successfully used in colorectal cancer with peritoneal carcinomatosis [9, 10, 13]. Mitomycin-C, and oxaliplatin are non-cell-specific drugs and may be used during HIPEC [20]. 5-FU is a chemotherapeutic drug that acts on G_2_ phase of the cell cycle and can be used only in EPIC [20].

The most frequent and serious complication was anastomotic leak (8.2%). The incidence was higher than the 5% postoperative leaks reported in common elective surgery [21]. Anastomotic healing is adversely influenced by mitomycin-C [22]. Local hyperthermia has no adverse effect on anastomotic healing [23]. It is well known that patients receiving intraperitoneal chemotherapy are prone to infections, abscess formation, sepsis or wound dehiscence [24]. Although no wound dehiscence was observed, various infections were quite frequent, and as a consequence the high incidence of postoperative complications is easily explained. EPIC, but not HIPEC, was found to be related to the increased morbidity, but it was not shown to be a prognostic indicator. Probably, the rapid infusion of the cytotoxic drugs into the peritoneal cavity has a negative effect on the recently performed anastomosis, which may be another explanation for the anastomotic failures and the enterocutaneous fistulas. Another explanation about the high rate of anastomotic leaks after EPIC is that the sutures of the digestive track are bathed for 5 consecutive days in a liquid of chemotherapy that makes them vulnerable [25]. The respiratory complications were also frequent (8.2%) and this has been reported by others [24].

Toxicity of intraperitoneal chemotherapy is very rare and mild if one drug is used for perfusion [24, 26]. The slowly absorbed cytotoxic drugs do not show high concentrations in the systemic circulation and cannot easily produce systemic side effects [18]. From this point of view, intraperitoneal chemotherapy seems to be superior to systemic chemotherapy. Only one patient in the EPIC group was recorded with mild neutropenia that did not require special treatment. With intraperitoneal chemotherapy renal toxicity is also avoided with close monitoring of the diuresis [19]. A very rare complication attributed to intraperitoneal chemotherapy is mild pancreatitis which was recorded in only one patient [27]. 

 The most optimistic report about the overall 5-year survival rate recorded 60% and 56% for colon and rectal cancer, respectively [28]. The difference in survival between the two groups is significant and may be due to either the difference in the degree of differentiation or the route of intraperitoneal chemotherapy (intraoperative versus postoperative) which has not been clarified. Similar, but not statistically significant difference in overall survival has been reported for colorectal cancer with peritoneal carcinomatosis [25]. In this paper the HIPEC group had better survival than the EPIC group. Although the 5-year followup has not been completed, no death related to cancer recurrence has been recorded in the HIPEC-group. Intraperitoneal chemotherapy has been shown to be related to survival, but it has not been proved to influence survival independently. However, it appears to play a significant role in the development of recurrence, as has been shown by multivariate analysis. It is of significant importance that the number of locoregional recurrences is very small. No patient in the HIPEC-group get 4 patients in the EPIC group developed locoregional recurrences. It is important to note that significant difference in the rate of recurrence has been documented for colorectal cancer with peritoneal carcinomatosis [25]. Patients that received EPIC developed recurrence more frequently than those that received HIPEC.

## 5. Conclusions

Although the number of included patients is small and further study is required, the use of intraperitoneal chemotherapy in the treatment of locally advanced colorectal cancer appears to play a significant role in reducing the incidence of recurrences. HIPEC seems to be more effective than EPIC. The survival is higher, and the number of recurrences is smaller with the use of HIPEC.

## Figures and Tables

**Table 1:** General characteristics.

Variable	HIPEC	EPIC	*P* value
Mean age	67 ± 13.2 (25–84)	70.9 ± 9.9 (38–92)	>.05
M/F ratio	25/15	33/24	>.05
Performance status			
90–100%/70–80%/50–60%	22/15/3	52/5/0	<.001
T (T_3_/T_4_)	35/15	50/7	>.05
N (N_0_/N_1_/N_2_)	20/14/6	31/15/11	>.05
Stage (II/III/IV)	19/19/2	31/26/0	>.05
G (G_1_/G_2_/G_3_)	11/25/4	3/49/5	.006
Anatomic distribution			>.05
Right colon	15	19	
Left colon	9	20	
Rectum	16	18	
Surgery			>.05
Right colectomy	15	16	
Transverse colectomy	0	3	
Left colectomy	9	22	
Low anterior resection	10	8	
Abdominoperineal resection	6	8	
Hospital mortality	1	9	.009
Morbidity	16	22	>.05
Recurrence	1	16	.001
Pattern of recurrence			
Distant/locoregional	1/0	12/4	>.05

**Table 2:** Postoperative complications.

Complication	HIPEC	EPIC	Total	Total %
Anastomotic failure	4	4	8	8.2
Respiratory	3	5	8	8.2
Wound infection	4	1	5	5.2
Enterocutaneous fistula	2	2	4	4.1
Sepsis	1	2	3	3.1
Cardiovascular	0	2	2	2.1
Acute renal failure	1	2	3	3.1
Postoperative bleeding	1	0	1	1
Intra-abdominal abscess	1	0	1	1
Urine infection	0	1	1	1
Pancreatitis	0	1	1	1
Pulmonary embolism	0	1	1	1
Neutropenia	0	1	1	1
